# Interplay between Carbonic Anhydrases and Metallothioneins: Structural Control of Metalation

**DOI:** 10.3390/ijms21165697

**Published:** 2020-08-09

**Authors:** Daisy L. Wong, Amelia T. Yuan, Natalie C. Korkola, Martin J. Stillman

**Affiliations:** Department of Chemistry, The University of Western Ontario, 1151 Richmond St., London, ON N6A5B7, Canada; dwong232@uwo.ca (D.L.W.); ayuan26@uwo.ca (A.T.Y.); nkorkola@uwo.ca (N.C.K.)

**Keywords:** metallothioneins, carbonic anhydrase, protein–protein interactions, electrospray ionization mass spectrometry, metalation kinetics, metal donation, pH dependence

## Abstract

Carbonic anhydrases (CAs) and metallothioneins (MTs) are both families of zinc metalloproteins central to life, however, they coordinate and interact with their Zn^2+^ ion cofactors in completely different ways. CAs and MTs are highly sensitive to the cellular environment and play key roles in maintaining cellular homeostasis. In addition, CAs and MTs have multiple isoforms with differentiated regulation. This review discusses current literature regarding these two families of metalloproteins in carcinogenesis, with a dialogue on the association of these two ubiquitous proteins in vitro in the context of metalation. Metalation of CA by Zn-MT and Cd-MT is described. Evidence for protein–protein interactions is introduced from changes in metalation profiles of MT from electrospray ionization mass spectrometry and the metalation rate from stopped-flow kinetics. The implications on cellular control of pH and metal donation is also discussed in the context of diseased states.

## 1. Introduction

Metalloproteins and metalloenzymes play key roles in cellular regulation, whether by catalysis of metabolites to maintain homeostasis (metabolomics), controlling metal ion buffering (metallomics, and related metalloproteomics), or by activating signaling pathways (metallotranscriptomics) [1,2]. As a fundamental step in performing their cellular roles, these proteins must interact with specific orientations to function [3]. As such, solution-phase, three-dimensional, protein–protein interactions are of vital interest, as their activity dictates life, and can potentially be harnessed to catalyze chemical reactions, often in stereospecific ways [4,5]. These protein–protein interactions are dependent on the correct folding of proteins and, therefore, metal-based status can lead to both refolding and unfolding.

Both the carbonic anhydrases and the metallothioneins are metalloprotein families that coordinate Zn^2+^ ions. While both classes of families are necessary for mitigating stress responses, the bioinorganic chemistry of these proteins with respect to the Zn^2+^ ion is distinct. Specifically, metallothionein (MT) is a Zn^2+^ storage protein that binds up to 7 Zn^2+^ with a Cys_4_ coordination, whereas carbonic anhydrase (CA) is dependent on a single Zn^2+^ ion with a His_3_ and H_2_O coordination primed for hydrolytic reactivity. MTs donate Zn^2+^ ions to apo-CA through protein–protein interactions, restoring CA catalytic activity [6]. The necessary protein–protein interactions suggest that MT’s role is as a highly specialized metallochaperone that minimizes competitive replacement of Zn^2+^ by toxic metal ions like Cd^2+^. Therefore, the metallothionein family, as a regulator of the metallome and, therefore, the cytoplasmic and extracellular environment, will indirectly affect the activity of the carbonic anhydrase family, and all other related cellular signaling cascades, including apoptosis [7].

**Figure 1:**
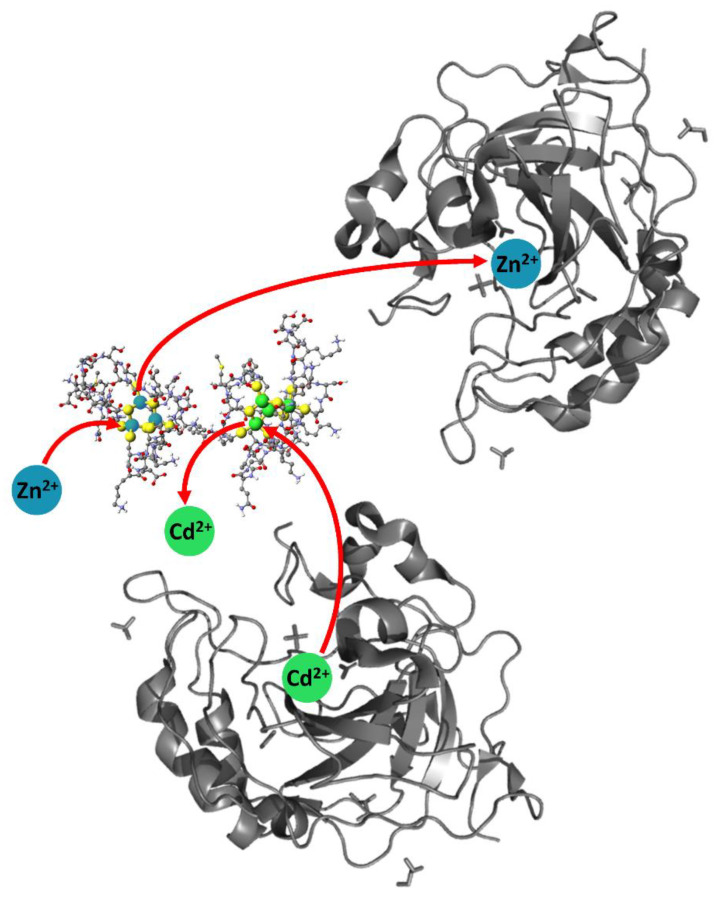
Molecular model of mammalian metallothionein 1A (ball and stick model) with cysteine sulfurs highlighted in yellow, essential mineral Zn^2+^ in blue, and toxic Cd^2+^ in green. Metallothionein (MT) interacts with human carbonic anhydrase (CA) (II) (ribbon models, Royal Society of Chemistry Protein Data Bank ID: 2VVB), one of many physiologically possible metallated states, Zn_3_Cd_4_-MT, where the thermodynamically favored Cd_4_S_CYS-11_ cluster forms to abstract toxic Cd^2+^. The Zn_3_S_CYS-9_ metal thiolate cluster in the N-terminal β- domain is more labile, with lower binding association K_a_ values than that of the Cd_4_S_CYS-11_, and is able to donate Zn^2+^ to inactive apo-enzymes.

This review first introduces both CA and MT (Section 1), followed by a description of the isoform-specific roles in cancers, in Section 2. Section 3 describes how CA and MT modulate cellular conditions, particularly environmental pH and zinc status and the consequence of cadmium toxicity. Section 4 highlights electrospray ionization mass spectrometric (ESI-MS) studies of zinc binding to CAs through both kinetic and equilibrium results. The change in the binding constants for apo-MT binding up to 7 zinc ions is interpreted in terms of protein–protein interactions (Section 4.1). Section 4.6 summarizes results with both electrospray ionization mass spectrometer (ESI-MS) experiments and stopped-flow kinetic studies of the metalation process involving cadmium, in which CA–MT association is demonstrated through the interferences of Cd metallation of MT before apo-CA is metallated (Figure 1). Moving forward from the foundations of ligand–protein interactions, we focus on the extension of methods that exploit the electrospray ionization mass spectrometer (ESI-MS) as a quantitative tool to determine biochemical interactions of protein mixtures.

### 1.1. Carbonic Anhydrases

Carbonic anhydrases (CAs) are omnipresent in living organisms. At approximately 30 kDa, this family of enzymes have a rigid secondary structure marked with 6 alpha helices and 10 beta pleated sheets [8]. This crucial catalyst controls the balance of CO_2_, bicarbonate (HCO_3_^-^), and water, affecting cellular pH, osmotic pressures, Ca^2+^ and Na^+^ transport, and neuronal signaling, among others [9]. Since their discovery in 1932, carbonic anhydrases are now staple model metalloproteins, with distinct structural features, high thermal stability, and well-defined crystal structure [10,11]. The Zn^2+^ ion is an essential cofactor, coordinated by His94, His96, and His119, with the fourth ligand of the near-tetrahedral coordination site being water [9]. The importance of the metal being Zn^2+^ cannot be overstated because of the strength of its Lewis acidity in activating the deprotonation of water at pH 7.4. Unlike MTs, the Zn^2+^ in CA is coordinated to the more electron withdrawing histidine residues. The selection of these coordinating amino acids tunes the reactivity of the electron-deficient Zn^2+^ ion to activate the water molecule. The resultant hydroxide anion is the catalytic key for the production of bicarbonate from CO_2_ and H_2_O in muscle tissue, as well as the reverse reaction in the lungs, acting as an anhydrase, a hydratase, and in some forms, as an esterase [9]. Because of CA’s ability to transform the metabolome and control the osmotic pressure in cells, CAs dynamically change the cellular environment, both intracellularly and extracellularly [12].

There are 7 genetically unique families of CAs, named α-, β-, γ-, δ-, ζ-, η-, and θ-CAs [13], of which the δ and ζ classes of CA are both expressed in marine diatoms [14]. In humans, a total of 15 active α-class isoforms have now been identified [15], along with 3 pseudogenes lacking the Zn^2+^ ion, rendering them inactive [16,17]. Isoforms are classified by structural similarities and have differential expression in organ tissue and subcellular localization. Briefly, CA I, CA II, CA III, CA VII, CA VIII, CA X, CA XI, and CA XIII are cytosolic, CA IV, CA IX, CA XII, and CA XIV are membrane-bound, CA Va and CA Vb are mitochondrial, and CA VI is secreted mainly through saliva, as well as milk and tears [15]. The most enzymatically active [13] and ubiquitous in humans is CA II, present in all tissues and essential for bone resorption and renal tubule resorption of bicarbonate. Further, in this review, the metalation studies discussed with MT involve CA II, which is also prominent in renal tissue [18], where CA II regulates intracellular pH.

The specific function of CA is defined by its location. CA is now known to affect the ocular, renal, skeletal, and central nervous systems, with large classes of inhibitors developed to treat diseases in such areas [19]. Particularly, sulfonamides received attention as blanket inhibitors that bind to the minor hydrophobic face of CAs. Acetazolamide is one such popular inhibitor, used to treat glaucoma, altitude sickness, and epilepsy, amongst others [9].

Given that CAs are widely studied and their structural, chemical, and physiological features have been thoroughly reviewed, we direct the reader to the many recent reviews in the bibliography [9,10,20,21,22,23], including those by Supuran [7,11,24,25,26]. This present review focuses on research regarding CA’s interactions with MT, a Zn^2+^ protein considered as a source of Zn^2+^ for activation of CAs. MTs may also compete for Cd^2+^, a carcinogen and deactivator of CA enzymatic properties. In that model, MTs protect CAs from the dangers of binding to cadmium.

### 1.2. Metallothioneins

Metallothioneins (MTs) are small proteins (6–8 kDa, depending on the degree of metalation) whose primary sequence is approximately 30% cysteine residues. Key characteristics include a lack of aromatic acid residues, and, despite the 20 cysteines in mammalian MT, a complete lack of disulfide bond formation. The cysteine thiols are the key moiety for metal binding, and MTs act as a major Zn^2+^ and Cu^+^ ion chaperone and metal ion storage protein in the cell. MT expression can be triggered by a variety of stresses, including metal exposure, glucocorticoids, inflammation, and oxidative stress [27]. As a result, MTs mitigate a variety of responses to stresses, and are a necessary thiol source for maintaining metal ion and reactive oxygen species homeostasis. MTs are key in activating Zn-dependent enzymes, such as CAs [28,29]. MTs are also implicated in other intracellular biological complexes, such as adenosine triphosphate (ATP) [30], and are implicated in direct protein interaction with caeruloplasmin [31] and ferritin [32], as well as bovine serum albumin [33,34].

In the cytosol, mammalian MTs can interfere with drug metabolism. This is achieved either through direct competition with the intended target, or through mediation of toxic effects by MT’s antioxidant capabilities [35]. Notably, MTs are heavily implicated in drug-resistance [36], particularly with the cancer-therapeutic *cis-*diamminodichloro platinum(II), known as cisplatin [37,38]. The Pt-ligand complex is broken down, and the native Zn^2+^ is released into the cytoplasm, triggering metal response elements (MREs) to signal for MT production [39,40]. Additionally, therapeutic alkylating agents can also induce MT-driven drug resistance, especially if antioxidant or glucocorticoid response elements are triggered [41]. While MTs are a large class that are found in all families of life in a variety of heterologous forms [42,43], given the focus of this review on isoform dysregulation in disease, we only discuss the highly conserved human MT isoforms. Plant, echinoderm, and other eukaryotic metallothioneins, however, are diverse and multi-faceted, with species having bespoke protein sequences tuned for a variety of metal-thiolate cluster formations [44,45,46,47,48,49,50,51,52,53].

**Figure 2:**
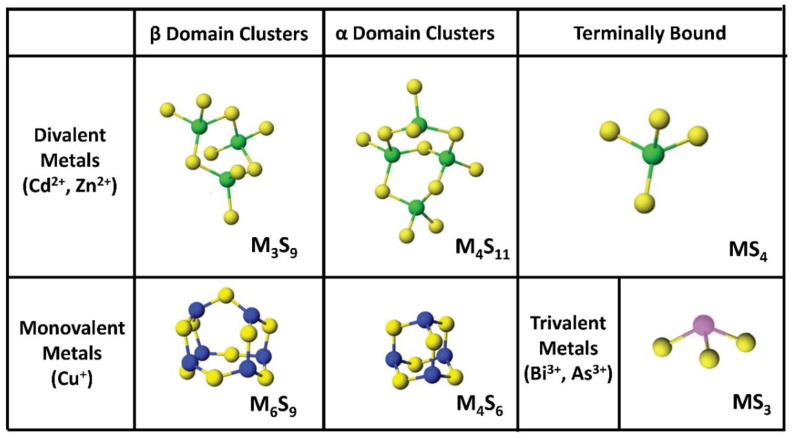
Binding motifs of mammalian metallothioneins. Ball and stick structures depict MT binding monovalent, divalent, and trivalent metals (blue, green, and pink, respectively) as clusters in the β and α domains and as terminally bound to the cysteine thiols (yellow). Adapted from References [57,58,59,60,61]. Excluded are plant metallothionein clusters, which are significantly more varied in structure [45].

MTs are unusual due to their metalation properties, which can result in a unique series of metal thiolate clusters that are very stable [54,55]. Examples of such multidentate coordination geometries are shown in Figure 2. Metalation induces specific protein folding that plays a role in metal donation to enzymes. Often described as cooperative, the formation of these thermodynamically stable clusters involve intricate metal-sulfur networks and geometries. We note that there are two distinct coordination patterns of MTs to metals, the first being the cooperative cluster formation, and the second being the initial tetrahedral cysteine chelation event of the metal ion. In a non-cooperative fashion, metal ions bind initially with the mono-coordinate cysteine thiols as M^II^S_CYS-4_, until all available cysteines are saturated. With increasing metalation and decreasing free cysteine availability, the cooperative cluster mechanism becomes favored. MT metalation is complicated because the flexibility of the peptide can be influenced by pH, metal identity, and temperature [56].

## 2. Mammalian Isoforms Associated with Dysregulation in Cancer

MTs and CAs are both large protein families with a kaleidoscopic range of isomers. The prevalence of isomers is location-specific. The change in expression of specific isoforms is often used as a biomarker for disease, as will be discussed below. Any change in expression greatly shifts the dynamic environment of cells, with downstream systems being affected as well. MT and CA are directly related in that MT will activate CA with donation of a Zn^2+^ cofactor [62,63]. Studies have also shown that apo-metallothioneins are capable of extracting Zn^2+^ and Cd^2+^ from CA more efficiently than ethylenediaminetetraacetic acid (EDTAH_4_) or pyridine 2,6-dicarboxylic acid [64]. In this section, we describe isoforms with noted changes in expression, but we stress that especially with MTs, the high sequence homology and lack of appropriate antibodies make complete characterization still an uncharted area.

### 2.1. Carbonic Anhydrase IX and XII

The many isoforms of CAs are encoded on multiple chromosomes, with CA I, CA II, CA III, and CA XIII all encoded on the same region of chromosome 8 (8q21.2). CA isozymes are specified by their cellular localization, distribution in organs and tissues, and expression levels, among other characteristics well-described in Reference [65]. Of note, in mammalian CAs, CA IX and CA XII usually have limited expression in healthy tissue, mostly restricted to the gastrointestinal tract and renal system [66,67]. This isozyme expression is associated with tumorigenesis. For solid tumors, CA IX and XII are situated on the extracellular matrix boundary in an abnormally low pH environment. These membrane-specific CA isoforms have a 75 amino acid long proteoglycan region (PG) that is specific to these tumorigenic CAs [68]. This proteoglycan-like domain maintains protein stability through an extensive mannose-type glycan network [69]. The PG region of CA IX is responsive to changes in extracellular pH and structurally transforms the active site, closed under normoxic conditions but open and available for catalytic activity and hydration of CO_2_ under hypoxic conditions [70].

These CA isoforms are homodimeric, anchored to the cell through a hydrophobic transmembrane region, with the dimeric catalytic region protruding into the extracellular matrix (ECM), increasing their overall catalysis efficiency and stability. CA IX, in particular, is a hypoxic marker for tumors, as it is strongly transcriptionally activated by hypoxia inducible factor-1 (HIF-1) [71]. When active, CA IX is as catalytically active as CA II, one of the most common CAs in humans [71]. CA XII, on the other hand, is a possible marker for chemotherapeutic resistance as it provides the optimal pH for multi-drug resistant factor, P-glycoprotein [72].

### 2.2. Mammalian Metallothioneins

Human MTs are encoded by a group of genes on chromosome 16q13, with four major isoforms. MT1 and MT2 (sometimes referred to as MT-2A, but this is equivalent) are basally expressed but highly inducible by metal ions and the stimuli discussed above. MT3 and MT4 are constitutively expressed in mainly the brain and epithelial cells respectively, and have no reported subisoforms. All isoforms contain 20 conserved cysteine residues, with the non-cysteine amino acids defining protein identity. The lack of differentiation between sequences in the isoforms has been a limiting factor in the development of (sub)isoform-specific antibody identification.

The differential expression of isoforms in relation to tumor growth has been suggested as a target for diagnostics or therapies [73]. Of these isoforms, the sub-isoform family of MT1 is the largest: with 13 sub-isoforms. MT-1A, -1B, -1E, -1F, -1G, -1H, -1M, and -1X are active genes, while there are 5 pseudogenes that are not known to form protein products in vitro (MT-1C, -1D, -1I, -1J, and -1L) [74,75]. There is no all-encompassing pattern for isoform expression in cancers, and changes in MT expression are cancer-specific.

Increased MT1 and MT2 mRNA and protein expression is observed in breast, kidney, lung, nasopharynx, ovary, prostate, salivary gland, testes, urinary bladder, cervical, endometrial, skin carcinoma, melanoma, acute lymphoblastic leukemia, and pancreatic cancers [76]. However, MT1 and 2 are downregulated in hepatocellular, gastric, colorectal, central nervous system, and thyroid cancers [76]. In breast cancers, MT2 expression is correlated with breast tissue growth, cell proliferation, and histological grade, while MT3 overexpression is associated with poor prognosis [77]. In renal cancers, MT2 upregulation and downregulation of MT-1A and -1G are observed, and in papillary thyroid cancer, upregulation of MT1 and MT2 is reported [77]. Downregulation of MT2 is recorded in nasopharyngeal cancer [78]. Curiously, MT3 is found to be upregulated in human bladder cancer, where it is not normally expressed [79]. MT-1X upregulation is implicated in the inhibition of hepatocellular tumor progression [80]. Downregulation of MT-1E has been observed in hepatocellular and prostate cancers [81] and may participate in alternative pathways that mimic the function of estrogen [77]. All these different channels of dysregulation influence the metallome and disrupt cellular growth signaling responsible for normal cell proliferation.

## 3. Cellular Control and Dysfunction

### 3.1. pH Effect on Protein Activity

Changes in pH affect all aspects of proteomic activity. Cancerous cells characteristically have a pH gradient across the extracellular matrix (ECM) that is reversed from normal cells [82]. Under these conditions, the extracellular matrix is significantly more acidic, promoting hypoxia, with values as low as pH 6.2 [83]. Intracellularly, the cytosolic pH increases from 7.2 to 7.4, which amplifies this pH imbalance, a characteristic of the Warburg effect. These conditions are established through the imbalance of metabolic processes, where anaerobic metabolism is dominant over aerobic metabolism, creating fermentation products such as ethanol and lactic acid [84]. These unusual conditions negatively affect the normal cells and their surrounding environment, giving cancerous cells an even greater growth advantage due to lack of competition with its non-cancerous counterparts [84].

CA controls cellular pH by maintaining the balance of bicarbonate and water, which can also be influenced by CO_2_ fluctuations in the cell. While the overall protein structure does not change drastically with a change in pH, the geometry of the active site can. For human CA II in the cytoplasm, the key proton acceptor His 64 that is located at the apex of the active site can shift its orientation, which allows CA II to overcome the rate-limiting proton-transfer step from the active site water molecule at high buffer conditions [85].

The hypoxic tumor environment upregulates many antioxidant proteins through HIF-1, such as glutathione-S-transferases, MTs, and CA IX [70,86,87]. Metallothioneins exhibit pH-dependent metal binding via the aforementioned cooperative and non-cooperative pathways. The pH range 6–8 encompasses the turning point in this coordination behavior, making MT sensitive to small changes over this range [58]. The low pH hypoxic conditions favor cluster formation of M^2+^ ions in MTs.

### 3.2. The Neccessity of Zinc

Zinc ions are essential to the chemistry in CAs, as well as in over 3000 other enzymes, transcription factors, and regulatory proteins [88]. These zinc proteins are divided into two general classes: (i) enzymes with a rigid zinc cofactor, and (ii) storage, chaperones, and transcription factors capable of rapid metal exchange and largely only found in eukaryotes [89]. It has been said about zinc, which is an essential nutrient for the chemistry of life, that it has “a finger in every conceivable pie”, being involved in all classes of enzymes [90]. As a result, dietary zinc deficiency can impact growth and cognitive function [91]. The Zinc transporter family of proteins are involved in Zn^2+^ trafficking and signaling, whose dysregulation by nutritional deficiency is suspected of disrupting zinc pathways in cancerous tissue. Examples of the effect of disruption includes childhood brain tumors [92], the activation of cell motility [93], or other activation cascades affected by Zn^2+^ signaling [94].

Zinc deficiency also influences absorption of other essential and toxic metals. For instance, a deficiency in zinc is directly related to differential copper absorption by MTs and ceruloplasmin in the liver [31]. Zinc absorption is also consequently disturbed by the absorption of other metals, like in the case of iron-rich diets [95]. Zinc-deficient organisms also experience increased absorption of Cd^2+^, which is of concern in food supplies grown in zinc-deficient soil (e.g., wheat [96] and rice [97,98]).

### 3.3. Interactions with Toxic Cd^2+^ and Xenobiotic Metals

Toxic Cd^2+^ ions can affect the native physiological chemistries of both CA and MT. Cd^2+^ is a Class I carcinogen according to the International Agency for Research on Cancer (IARC), and disrupts the renal, skeletal, and endocrine systems [99]. Cadmium causes toxicity and tumorigenesis through oxidative stress, inhibition of DNA repair mechanisms, and resistance to apoptosis, among others [100]. Cd^2+^ displacement of isoelectronic native Zn^2+^ in proteins and regulatory responses can cause often irreversible disruption to biological structures [90] and to native zinc and copper regulation [101].

For CAs, Cd^2+^ binds to the Zn^2+^ active site but this inhibits enzymatic function in mammalian CAs. There is evidence of an adaptive CA in marine diatoms (*Thalassiosira weissflogii*) that use Cd^2+^ in low Zn^2+^ environments, now considered a subclass of their own, ζ-CAs [102]. Otherwise, it is generally accepted that CA activity is virtually halted with Cd^2+^ replacement. This is true with most metals and CAs, with only Co^2+^ showing little enzymatic activity [103].

Metallothioneins are key players in the detoxification of Cd^2+^ in mammals, but the concentration and accumulation of Cd-MT in the kidneys for decades eventually leads to necrosis and kidney failure [104]. Currently, MTs are known to interfere with platinum-based anticancer drugs and doxorubicin treatment of breast cancer [105]. In vitro studies show MTs binding to xenobiotic metal drugs cisplatin [106,107,108] and therapeutically relevant ruthenium and rhodium complexes in unusually anarchistic ways [39,109,110,111]. Unlike CAs, xenobiotic metal replacement does not deactivate MT activity.

## 4. MT-CA Studies

### 4.1. Kinetic Analysis of Zn^2+^ Reactivation of Apo-CA

Early studies regarding the interactions between CA and MT focused on the transfer of Zn^2+^ from Zn-MT to apo-CA. The kinetics, pH dependence, and activity of the reconstituted enzyme were key observables in these studies. Of particular importance were the studies by Udom et al., which showed Zn-MT reactivation of apo-CA by detecting the renewed catalytic activity [62]. In the 1980′s, Petering et al. reported that the ligand exchange rates and the subsequent reconstitution of CA were significantly faster with Zn-MT than when the Zn^2+^ was complexed with ethylenediamine tetraacetic acid (to form Zn-EDTA). They proposed from their stopped-flow observations that MT efficiently associates with CA, setting up direct metal transfer via protein–protein interactions [63].

Huang et al. from Fudan University created synthetic Zn^2+^-thiolate clusters and compared their reconstitution of apo-CA to that by native Zn-MT, by monitoring the hydrolysis of p-nitrophenyl acetate. They demonstrated from their X-ray diffraction studies of the synthetic thiolate clusters that the metal-thiolate bond lengths in Zn-MT were longer than expected, and they proposed that this imparted lability on the zinc ion, assisting in donation [112]. Huang et al. link these results to their previous study of Zn-MT reconstitution of apo-CA by emphasizing the importance of the polypeptide backbone in the formation of the MT-CA protein-complex, which is a necessary step to weaken the Zn-S bonds, allowing for metal donation [113]. This was believed to be the rate determining step.

Different cluster structures result in different metal donating properties. By monitoring the rate of restored enzymatic activity, Shi et al. showed that Cu_4_Zn_3_-MT3 was more efficient at restoring CA activity than Zn_7_MT1, Zn_7_MT2, Cd_5_Zn_2_MT1, or Cd_5_Zn_2_MT2, likely due to the ~6–8 extra amino acids in the MT3 protein sequence [114]. The longer protein backbone of MT3 imparts greater flexibility, which plays a role in Cu/Zn swap in peptide aggregates in neuronal diseases [115,116].

### 4.2. Equilibrium Competition Studies of Zn^2+^ Binding between Apo-βα-Metallothionein and Apo-Carbonic Anhydrase Using ESI-MS Methods and Affinity Constant Simulations

The Fenselau group was a leader in the realm of ESI-MS analysis of metallothioneins. At the time, in the early 1990’s, mass spectrometry was being recognized as a high-resolution technique for analyzing protein complexes [117]. Fenselau and coworkers used ESI-MS to identify the many partially metallated species of MT that accompany metal titration, while showing the ease of use, especially in the case of direct solution infusion. Their work highlighted the sensitivities of metallation to the pH of the environment, suggesting the pH dependence of cooperativity that is now widely understood [118]. They also demonstrated the power of ESI-MS in drug–protein complex analysis [119]. In the 21st century, ESI-MS studies of metallothioneins have moved on to semi-quantitative and quantitative analysis of metal–protein stoichiometries. An excellent description of the concept and approach is provided by Blindauer [90].

**Scheme 1:**
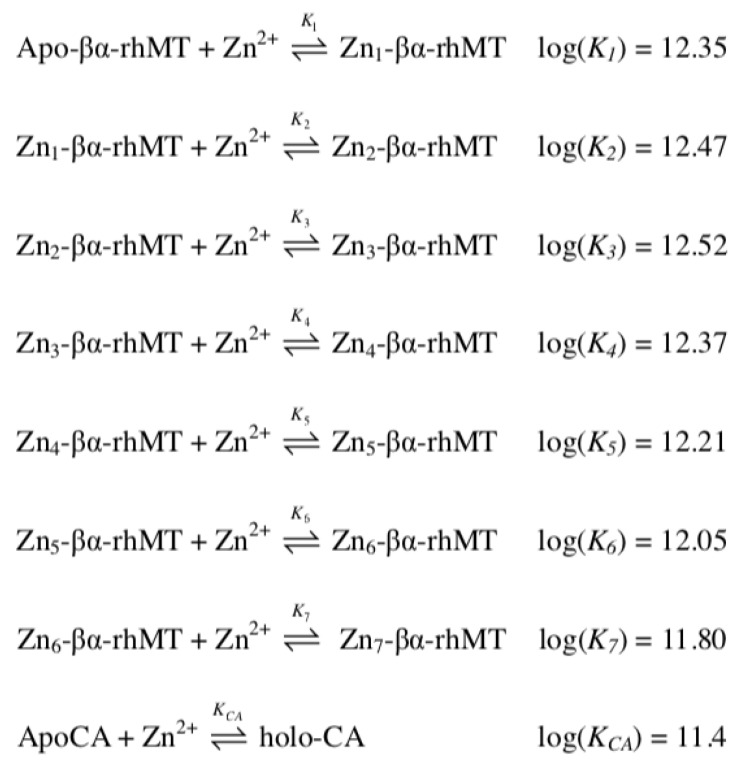
Competitive and sequential metalation reactions of apo-MT and apo-CA. “rhMT” refers to recombinant human MT. Reproduced with permission from American Chemical Society, 2014, Reference [120].

**Figure 3:**
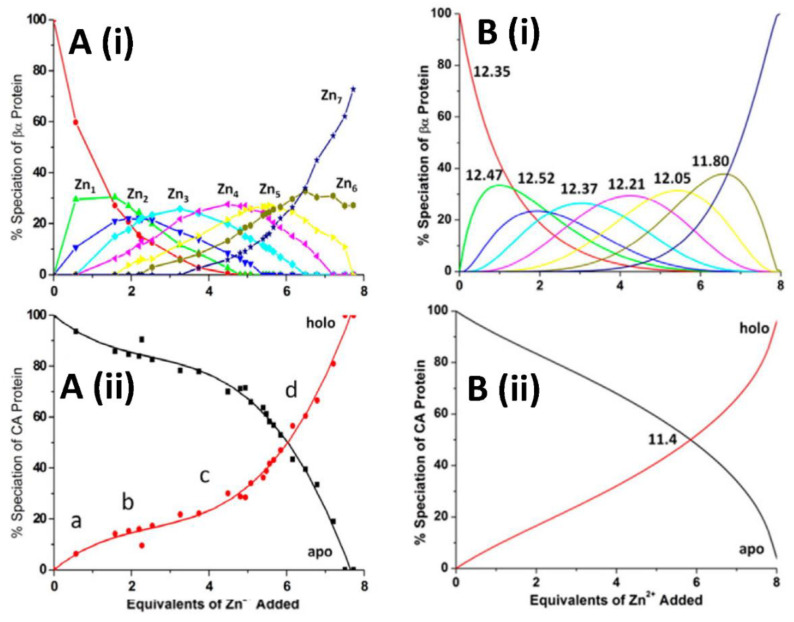
**A**(**i**) Experimentally determined and simulated **B**(**i**) competitive zinc metallation of apo-MT with the sequential metallation steps represented by colored lines: apo-MT (red), Zn_1_ (green), Zn_2_ (blue), Zn_3_ (cyan), Zn_4_ (magenta), Zn_5_ (yellow), Zn_6_ (olive), and Zn_7_ (navy). Experimentally determined **A**(**ii**) and simulated **B**(**ii**) competitive zinc metallation of apo-MT in the presence of apo-CA. Colored lines represent apo-CA (black) and holo-CA (red). Simulated log_10_(K_F_) values for MT were 12.35, 12.47, 12.52, 12.37, 12.21, 12.05, and 11.80 for the 7 sequential metalation events in the formation of Zn_7_MT, described in Scheme 1. These values were derived from extraction of the competitive speciation of the MT versus the known log_10_(K_F_) of CA of 11.4. Reproduced with permission from American Chemical Society, 2014, Reference [120].

The competition experiment in which apo-CA and apo-MT competed for Zn^2+^ reported by Pinter and Stillman allowed all 8 binding constants, K_1–7_ for apo-MT, and K_1_ for apo-CA, to be determined (Scheme 1) [120]. The use of ESI-MS methods quantified the metalation status of both proteins simultaneously and showed in detail the metal distribution, including the fraction of remaining apo-proteins, as a function of the mole equivalents of Zn^2+^ added [120,121]. Figure 3A(i) shows, based on experimental data assembled from multiple titrations, how the individual Zn^2+^ ions bind one by one in a completely non-cooperative pathway following the individual reactions in Scheme 1. Figure 3A(ii) shows the metalation status for the apo-CA as a function of stepwise additions of Zn^2+^ [58,122]. It is noteworthy that while the binding of a metal is usually straightforward to determine, including the apo-protein in the same data is often not possible. Clearly, apo-CA only metalates significantly after the stoichiometry of 5 Zn^2+^ per mole MT have bound.

The concurrent measurement of the metalation of the apo-MT and apo-CA allows the binding constant, K_1_, for Zn-CA to be used to calibrate the seven relative K_F_’s for Zn_1-7_-MT, Figure 3B(i). In this Figure, the simulation includes the concentrations of the apo-MT, apo-CA, and Zn^2+^ added in steps, and models the binding sequence and abundances of all 8 Zn^2+^. The important data point is that if log_10_(K_Zn_) = 11.40 for apo-CA plus Zn^2+^, then the 7th and last Zn^2+^ binding to the apo-MT binds with a larger K_F_ (of 11.80).

Inspection of the 7 MT K’s revealed an unusual trend. The K_1_ and K_2_ (log_10_ 12.35 and 12.47) are less than K_3_ (12.52), when usually the first metals to bind do so with the highest K_F_’s [123]. The log_10_K trend from K_3_ to K_7_ is linear with the number of Zn^2+^ ions bound, as expected for the statistical reduction in sites and without noticeable cooperativity at this pH. The interpretation at the time was that as this trend was very different than reported for Zn^2+^ metalation of free apo-MT, there had to be some form of protein–protein interaction between the MT and CA. This important aspect of the metalation of apo-MT is discussed in Section 4.4 below.

### 4.3. Equilibrium Competition Studies of Zn^2+^ Binding between α-MT and β-MT Fragments and Apo-Carbonic Anhydrase Using ESI-MS Methods and Affinity Constant Simulations

The Zn^2+^ competition between the full apo-βα-MT and apo-CA described above clearly showed that CA only metalated when MT had bound 4-5 Zn^2+^, indicating the accessibility of the 6th and 7th Zn^2+^ ions bound, as previously reported [120]. Our group had observed unusual As^3+^ metalation behavior of the fragments, where in the absence of a cooperative cluster formation, the magnitude of K values decreased linearly, as if they were 6 equally equivalent sites [59,123]. To probe the significance of the two-domain structure of Zn_7_-MT in the transfer of Zn^2+^ to apo-CA, the α-domain fragment and β-fragment competed for Zn^2+^ in the presence of apo-CA. As in Section 4.2, the sequence of metalation speciation for the individual domains of MT is described as a series of bimolecular reactions, their relative populations collected over series of titrations by ESI-MS, and the data fitted to provide individual binding constants (K_1–4_ for α-MT and K_1–3_ for β-MT).

**Figure 4:**
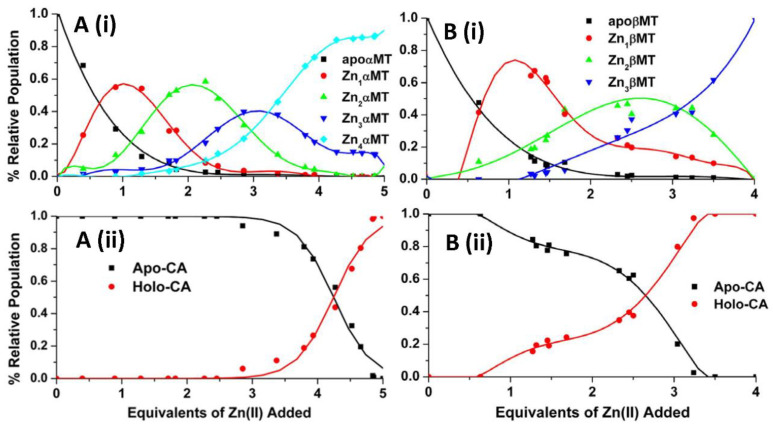
(**A**) Experimentally determined speciation of the competitive zinc metalation of apo-αMT domain fragment **A**(**i**) in the presence of apo-CA, and **A**(**ii**) extracted experimental speciation profiles from deconvoluted electrospray ionization (ESI)-mass spectral data, recorded during the competitive zinc titration of equimolar mixtures of apo-αMT and apo-CA at pH 7.0. Sequential metallation steps are represented by coloured lines **A**(**i**): apo-MT (black), Zn_1_ (red), Zn_2_ (green), Zn_3_ (blue), and Zn_4_ (cyan). The fraction of each species has been plotted according to the stoichiometry of the zinc added. An equivalent means zinc added for one metal-binding site. The lines are the predicted speciation based on the series of fitted K_F_’s shown graphically in Figure 5. Reproduced with permission from American Chemical Society, 2015, Reference [121]. **B**(**i**) Experimentally determined speciation of the competitive zinc metalation of apo-βMT domain fragment in the presence of apo-CA (black lines) and the subsequent formation of holo-CA (red lines). **B**(**ii**) Extracted experimental speciation profiles from the deconvoluted ESI-mass spectral data recorded during the competitive zinc titrations of equimolar mixtures of apo-βMT and apo-CA at pH 7.0. The fractional presence of each species has been plotted according to the stoichiometry of the zinc added. An equivalent means one metal-binding site. The lines have no theoretical significance. Reproduced with permission from Portland Press, Reference [121].

The C-terminal α-domain fragment at maximum capacity binds four Zn^2+^ ions in a Zn_4_S_CYS-11_ cluster involving a bridging thiolate-metal network, as described before. The ESI-mass spectral data in Figure 4A(i), represented in terms of speciation as a function of added Zn^2+^, shows a straightforward series of metalation steps with the calculated log_10_ K’s of the four steps for Zn_1-4_-α MT at 13.45, 13.15, 12.70, and 12.56, being an order of magnitude greater than the corresponding value of 11.40 for Zn_1_-CA. As we will introduce below, the trend in these four K_n_ (*n* =1–4) values follows the statistical availability of the 4 sites in the α-domain-fragment with little noticeable deviation from a linear negative slope.

The N-terminus β-domain fragment binds a maximum of three Zn^2+^ ions in a cluster with a stoichiometry of Zn_3_S_CYS-9_. The significance of the onset of this cluster formation is that the Zn^2+^ binding site in the β-MT changes from a tetrahedral structure based on four terminally attached cysteinyl thiolates (ZnS_CYS-4_), to the same tetrahedral geometry but involving bridging thiolates in Zn_3_S_CYS-9_. This is clear experimental evidence for the simulation-calculated values for the log_10_K’s of 12.24, 11.74, and 11.42 for the apo-β-MT compared again with the 11.40 for the apo-CA. The ESI-mass spectral data reproduced in Figure 4B(i) and (ii) show that Zn^2+^ begins to bind to the apo-CA in parallel with the formation of the Zn_3_S_CYS-9_ cluster, whereas the Zn_4_S_CYS-11_ cluster of the α-fragment is formed prior to apo-CA metalation. While the transformation from ZnS_CYS-4_ to bridging Zn_3_S_CYS-9_ involves a similar protein rearrangement to the α-domain cluster formation, the binding constants are an order of magnitude less than the α-domain fragment, and overlap that of Zn^2+^ binding to apo-CA. In the context of the whole protein (βα-MT), the Zn^2+^ binding sites in the β-domain are more labile, and are the last to be metalated, and the first to be donated. The magnitude of the calculated K’s also fit the interpretation above concerning the first cluster that forms in the α-domain of the full MT protein.

**Figure 5:**
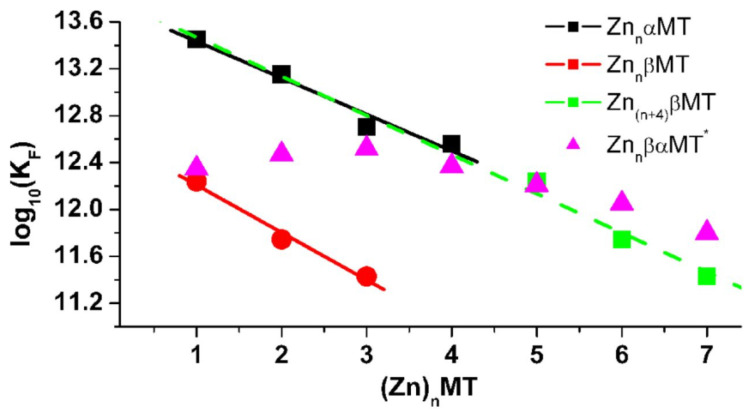
Comparison of the calculated zinc affinity constants for zinc binding to αMT1A (black) and βMT1A (red). The symbols show the calculated binding constants from the data in Figure 3 and Figure 4. The green dashed line shows the predicted linear trend for the β-fragment affinities shifted by four to illustrate the expected linear trend for connected fragments. * Affinity data for the zinc binding to the intact βαMT taken from Reference [120]. Reproduced with permission from Portland Press, Reference [121].

Figure 5 summarizes all 14 log_10_K_F_ values calculated from Zn^2+^ competition data between apo-MT (the two fragments and the whole protein) and apo-CA at pH 7.4. The green dashed line shows the 4 log_10_Ks for the 1–4 Zn^2+^ binding to the α-MT followed by 3 log_10_Ks for the β-MT shifted from 1–3 Zn^2+^ to 4–7 Zn^2+^ bound total. This virtual βα-MT displays a clear linear trend, giving a prediction of the trend for the whole protein if the isolated fragments were joined. However, and very significantly, the trend in log_10_K_F_ data is not at all linear for the 7 Zn^2+^ binding to the native apo-βα-MT in the presence of the apo-CA (Figure 5 above, pink triangles). Rather, the first three K_F_ values (for the metalation of the first 3 Zn^2+^ ions to apo-MT) are approximately the same, which goes against both the statistical trend, and the trend in the absence of apo-CA. The conclusion is that although apo-CA is not being metalated, the apo-MT metal-induced folding is impeded [56,124].

The initial steps in the metalation of apo-MT require the rearrangement of four cysteinyl thiols to form an isolated tetrahedral binding site for the Zn^2+^, summarized as [Zn(S_CYS_)_4_]^2-^. The subsequent Zn^2+^ ions added have to duplicate this process, and as described previously, up to 5 Zn^2+^ will form a string of these “beaded” structures, sequentially: first [Zn(S_CYS_)_4_]^2-^, then a second [Zn(S_CYS_)_4_]^2-^, etc. The K_F(n)_ for *n* = 1–3, indicate that the process of folding the peptide around each Zn^2+^ ion initially reduces the K_F_ by an order of magnitude from the expected value (10^12.4^ compared with 10^12.5^). This deviation from the green linear trend in Figure 5 is representative of the effect of apo-CA on the metalation of apo-MT. Therefore, there exists an association between the apo-CA and the apo-MT, such that the cysteinyl thiols are buried and not readily available for Zn^2+^ binding. The question of why the two α and β domain fragments metalate normally has not be directly probed, but we can suggest that this is due to the significantly shorter peptide length. The shorter length reduces protein–protein interactions, allowing the metalation to take place more readily without noticeable distortion of the normal, free-peptide values.

### 4.4. Kinetic Analysis of CA-MT: Zn^2+^ and Cd^2+^ Exchange Show Indirect Evidence of Protein–Protein Interactions

**Figure 6:**
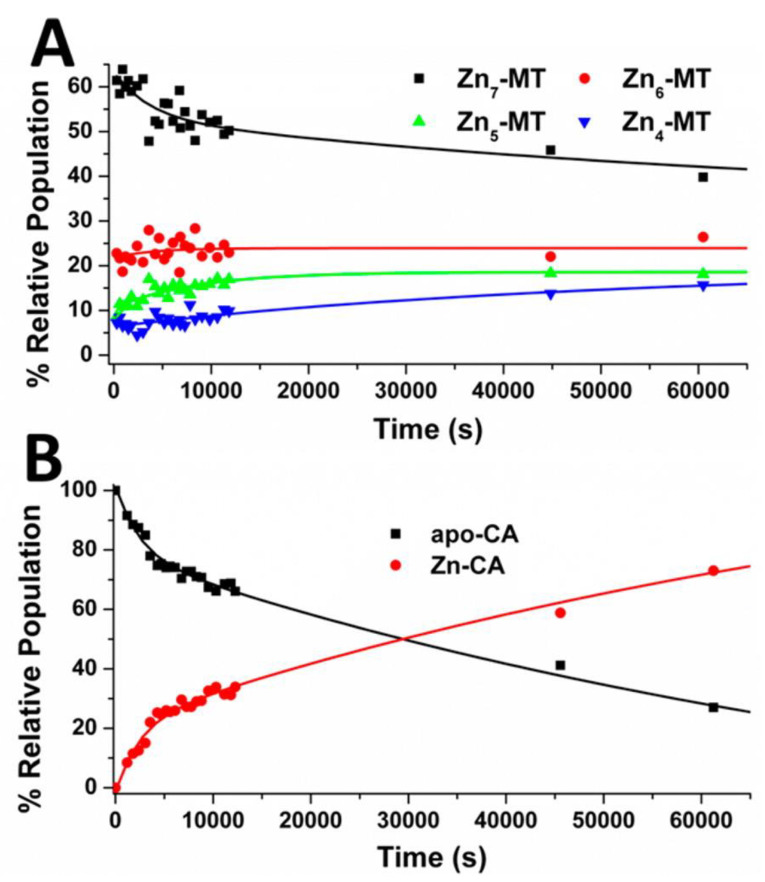
Time courses of the demetalation of (**A**) Zn-MT and the metalation of (**B**) apo-CA extracted from the ESI-mass spectral data. The lines are calculated from the fitted rate constant, k = 2.5 ± 0.5 M^−1^ s^−1^ (at 37 °C, pH 7.0). Reproduced with permission from American Chemical Society, 2015, Reference [125].

**Figure 7:**
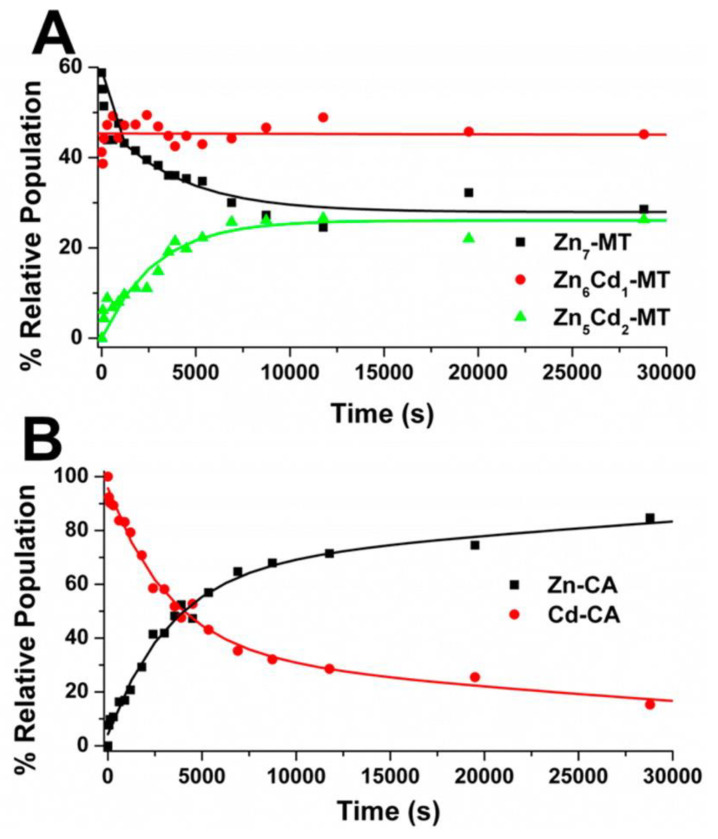
Time course of the metal exchange between Zn_7_-MT and Cd-CA. (**A**) MT and (**B**) CA. Species were extracted from the ESI-mass spectral data of the reaction between an equimolar (30 μM) mixture of Cd-CA and Zn_7_-MT. The lines are calculated from the fitted rate constant, k = 6.0 ± 0.5 M^−^^1^ s^−^^1^ (at 37 °C, pH 7.0). Conditions: 5 mM ammonium formate, pH 7.0, 37 °C. Reproduced with permission from American Chemical Society, 2015, Reference [125].

Simultaneous to the detailed ESI-MS data above, kinetic analysis of the speciation data can be obtained. We describe three situations studied to probe MT association properties with CA: (i) the rate of donation of Zn^2+^ from partially metalated Zn_n_MT (*n* = 1–7) to apo-CA, (ii) the exchange of Cd^2+^ in Cd-CA for Zn^2+^ from Zn_7_-MT (in effect, the scavenging of Cd^2+^ by Zn_7_MT), and (iii) the scavenging of Cd^2+^ in Cd-CA by partially metalated Zn_5_-MT. Figure 6; Figure 7 show the time course of these reactions based on time-dependent ESI-MS data, extending to 1000 min.

Figure 6 describes situation (i), quantifying the metal donation from saturated Zn_7_MT to apo-CA [125]. The speciation pattern is the same as that of the metalation of apo-MT at physiological pH [58]. Here, the ESI-MS experimental data taken from Figure 3A(i) and (ii) show that a distribution of Zn_n_ (*n* = 4, 5, 6, and 7) is formed by the demetallation by apo-CA. The key result was that these reactions are slow, which is generally considered unexpected with such large K_F_ values. The rate law is bimolecular, and dependent on both the apo-CA and Zn-MT concentrations. Therefore, to explain the large K_F_’s with the slow reaction rate, we reiterate the suggestion by Ejnik et al. regarding MT-CA protein–protein interaction: the rate-limiting step is the protein–protein association that facilitates metal donation.

The second set of data, in Figure 7, probes the exchange reaction between Cd-CA and Zn_7_-MT. The ESI-MS data provide details of each species. Again, the rate law is second order. The exchange of Zn^2+^ in Cd-CA is faster than Zn^2+^ insertion into apo-CA, which is unintuitive as there are no available binding sites. The Zn-Cd swap must occur through a different and more complicated mechanism compared with the metal exchange that takes place with apo-CA reconstitution. It likely involves a different rearrangement, bearing in mind the high K_F_ values associated with this reaction. This Zn-Cd swap may involve the formation of a transient super-metalated M^2+^_8_-MT species similar to that reported by Sutherland et al. [126].

### 4.5. Electrospray Ionization Mass Spectrometry Shows Indirect Evidence of MT-CA Interactions from Cd^2+^ Metalation Studies

**Figure 8:**
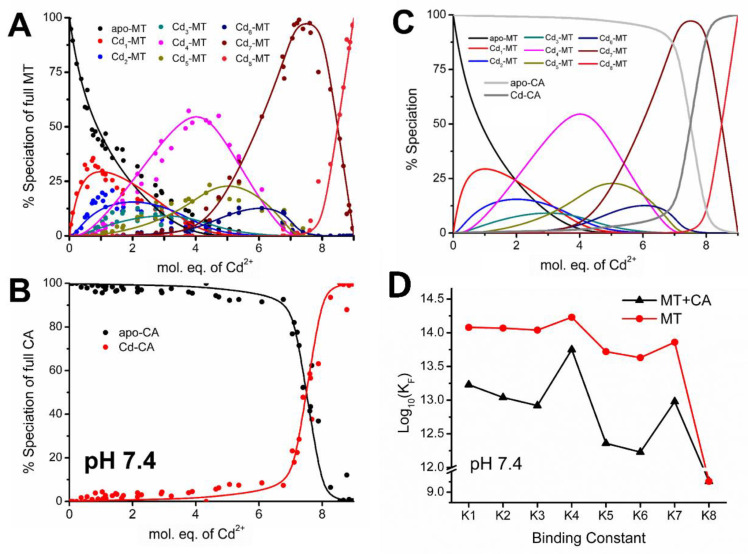
Experimental (dots) and simulated (lines) data extracted from the metal loading of (**A**) MT and (**B**) CA as a function of stepwise addition of Cd^2+^ at pH 7.4, based on ESI-MS data. The simulation model shown is one of 10 different simulations performed. The average log_10_(K_F_) values are 12.85, 12.75, 12.73, 13.47, 12.12, 12.14, 12.93, and 9.67 for the 8 sequential metalation events in the formation of super-metalated Cd_8_MT. The simulated data for CA is based on the reported log_10_(K_F_) value of 11.1. (**C**) Combined speciation model for metalation of apo-MT and apo-CA at pH 7.4. (**D**) The trend in log_10_(K_F_) values for the Cd^2+^ metalation of MT in the presence of CA (▲ MT + CA) and absence of CA (● MT) at pH 7.4. K_1_ → K_8_ are the equilibrium constants for the addition of 1 to 8 Cd^2+^ to MT. log_10_(K_F_) values for MT in the absence of CA were determined from a simulation model for ESI-MS data. The log_10_(K_F_) values for MT in the absence of CA were calibrated by setting the value for K_8_ to be the same as K_8_ in the competition data. log_10_(K_F_) values for MT in the absence of CA are 14.59, 14.53, 14.51, 14.75, 14.14, 14.13, 14.20, and 9.67 for 8 stepwise metalation events leading to Cd_8_–MT. The error bars represent the standard deviation of 10 different simulations of the data and the associated log_10_(K_F_) values. The log_10_(K_F_) values are significantly different for each stepwise addition from K_1_ → K_7_ and are different as an average of all log_10_(K_F_) values between MT in the absence and presence of CA. Reproduced with permission from Royal Society of Chemistry, 2020, Reference [127].

While Cd^2+^ is toxic, it is a spectroscopically more valuable probe, and provides a greater mass separation in the mass spectra than that of Zn^2+^. Simultaneous kinetic and ESI-MS studies of the Cd^2+^ metalation of apo-MT, isoform 1, were carried out using ESI-MS methods in the presence of apo-CA II at pH 7.4 and pH 5.0 by Yuan et al. [127]. These studies emphasize the pH-dependent association of the two proteins. With equimolar concentrations of apo-MT and apo-CA at pH 7.4, the metalation cascade of apo-MT is skewed due to the significant thermodynamic adjustments for cluster formation involving the K_4_ step. The Cd-S ligand to metal charge transfer band at 250 nm is emphasized in the cooperative cluster formation of Cd_4_S_CYS-11_. Compared to the lighter Zn_4_S_CYS-11_, the Cd-S bonds are comparatively more energetically stable because of the nephelauxetic effect, which explains its propensity to form at biologically relevant pH values. These studies confirmed the depression of the first K_F_ for Zn-binding to apo-MT in the presence of apo-CA, as described in Section 4.3, but with better clarity. Figure 8 shows the experimental ESI-MS data recast in the form of speciation as a function of added Cd^2+^.

Figure 8A shows the speciation for the apo-MT, aligned above the Cd^2+^ metalation status of the apo-CA in Figure 8B. Figure 8C shows the combined modeled speciation based on the calculated K_F_ values for both proteins. Even without the model, it is apparent that the Cd^2+^ metalation K_F_ for apo-CA is somewhat lower than the 7th Cd^2+^ binding constant, K_7_, to the apo-MT. These K_F_’s are plotted in Figure 8D as the lower line. What is clear is that there is nearly a 100-fold reduction in the value of the K_F_’s for the Cd^2+^ metalation of apo-MT in the presence of apo-CA. Note that the individual log K’s are listed in the Figure 8 caption.

A key and significant feature of these data is the prominence of the Cd_4_-MT species. At pH 7.4, in the absence of apo-CA, the value of K_4_ does not dominate to such an extent, compared with the value in the presence of apo-CA. In the presence of apo-CA during the metalation of apo-MT, the Cd_4_-MT cluster species forms instead of a non-cooperative CdS_CYS-4_ coordination. This cooperative K_4_ step highlights the importance of protection against mis-metalation, as compared to the K_4_ for Zn^2+^ binding seen in Figure 5, pink triangles. The depressed and skewed binding constants were determined through modelling of the speciation of MT observed by ESI-MS and known binding constant of Cd-CA.

**Figure 9:**
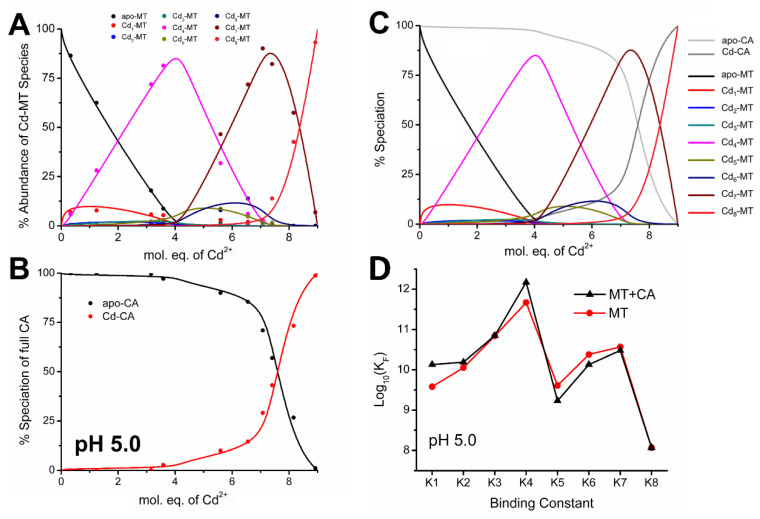
Experimental (dots) and simulated (lines) data extracted from the metal loading of MT and CA as a function of stepwise addition of Cd^2+^ at pH 5.0, based on ESI-MS data. (**A**) The simulation model uses log_10_(K_F_) values of 10.44, 10.49, 11.16, 12.49, 9.53, 10.30, 10.85, and 8.30 for the 8 sequential metalation events in the formation of super-metalated Cd_8_–MT. (**B**) The simulated data for CA is based on the reported log_10_(K_F_) value of 9.2. (**C**) Combined speciation model for metalation of apo-MT and apo-CA at pH 5.0. (**D**) The trend in log_10_(K_F_) values for the Cd^2+^ metalation of MT in the presence of CA (▲ MT + CA) and absence of CA (● MT) at pH 5.0. K_1_ → K_8_ are the equilibrium constants for the addition of 1 to 8 Cd^2+^ to MT. log_10_(K_F_) values for MT in the absence of CA were determined from simulation models for ESI-MS data. The log_10_(K_F_) values for MT in the absence of CA were calibrated by setting the value for K_8_ to be the same as K_8_ in the competition data. log_10_(K_F_) values for MT in the absence of CA are 9.65, 10.32, 10.67, 12.21, 9.46, 10.53, 10.69, and 8.30 for 8 stepwise metalation events leading to Cd_8_–MT. Reproduced with permission from Royal Society of Chemistry, 2020, Reference [127].

However, this phenomenon was not present when the pH was lowered to pH 5.0. Metalation of apo-MT in the presence of apo-CA at pH 5.0 showed that Cd^2+^ binding constants are the same in the presence and absence of apo-CA. Figure 9A shows the speciation from ESI-MS data for metalation at pH 5.0, aligned above the Cd^2+^ metalation status of the apo-CA in Figure 9B. The simulated values are overlapped in Figure 9C. The presence of the prominent Cd_4_-MT is also observed when metalating apo-MT in the absence of apo-CA. The key data are summarized in Figure 9D, where the alignment of the 8 K_F_’s of Cd^2+^ in the presence and absence of apo-CA are plotted. We interpret that this minimal change of K_F_’s results from the lack of a protein–protein structure at pH 5.0. Note that the individual log K’s are listed in the Figure 9 caption.

To understand in more detail the effects that were taking place when the apo-MT and apo-CA associated, we introduce the kinetics of the metalation process. Until recently, the kinetic analysis had not been able to shed light on the pathways followed when apo-MT metalated, but analysis of the Cd^2+^ metalation rate of the unfolded and folded apo-MT provided a powerful tool to probe changes [56]. Using stopped-flow kinetic methods, described in the following section, we had probed the effect of the presence of the apo-CA on the actual metalation events.

### 4.6. Changes in the Apo-MT Metalation Kinetics in the Presence of Apo-CA

Stopped-flow methods combined with UV-visible absorption spectroscopy were used to study the kinetics of Cd^2+^ binding to apo-MT in the presence and absence of CA at pH 5.0 and 7.4 to investigate the possible interactions of MT and CA seen in the ESI-MS results [127].

It has been shown that metalating apo-MT with 2.5 mole equivalents of Cd^2+^ will force a cluster pathway to form Cd_4_MT at pH 5.0 (Equation (1)) and a beaded pathway at pH 7.4 (Equation (2)) [56,58].

apo-MT + 2.5 Cd^2+^ → Cd_4_MT (1)

apo-MT + 2.5 Cd^2+^ → Cd_1_MT + Cd_2_MT + Cd_3_MT (2)

Previous studies on the kinetics of Cd^2+^ metalation of apo-MT have revealed that the bridging thiolate cluster structures form more slowly than the terminally bound beads [56]. Also, it has been shown that metalation will slow upon addition of a denaturant [56,128]. Following these results, it was hypothesized that other proteins in the cytoplasm interacting with the MT may also impede metalation.

**Figure 10:**
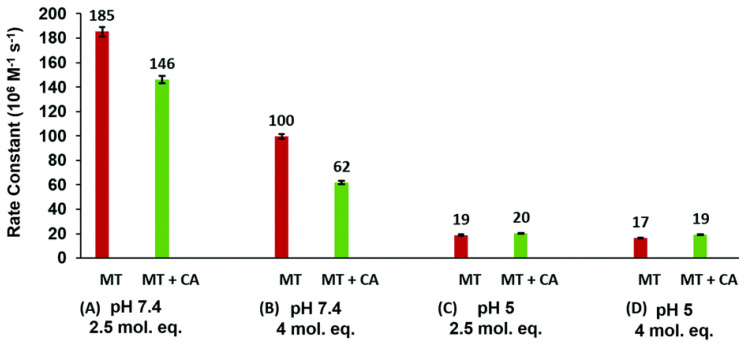
Comparison of the rates of Cd^2+^ metalation of apo-MT as a function of pH and the presence of apo-CA and various mole equivalents (mol. eq.) of Cd^2+^added (**A**,**B**) compare metalation at pH 7.4 show that the rate of Cd^2+^ metalation of apo-MT is significantly slower in the presence of apo-CA. (**C**,**D**) at pH 5.0 show no difference in metalation rate with or without apo-CA present. Reproduced with permission from Royal Society of Chemistry, 2020, Reference [127].

In our recent study, apo-MT was metalated at pH 5.0 and 7.4 using 2.5 and 4 mole equivalents of Cd^2+^ in the presence and absence of apo-CA [127]. The results show, through comparison of rate constants, that metalation of apo-MT with both 2.5 and 4 mole equivalents of Cd^2+^ is slowed down in the presence of CA at pH 7.4, when the beaded structures are predominantly forming. This suggests a possible interaction between MT and CA at this pH. At pH 5.0 however, this effect is not observed, and the differences between rates of apo-MT alone and with apo-CA were not significant. These rate constants have been summarized in Figure 10.

Because the rate slows when CA is present at pH 7.4, this supports the interpretation that MT is metalated through a clustered pathway under these conditions, as shown by the pattern of binding constants. The kinetic results ultimately correspond with the ESI-MS studies above, suggesting a possible interaction between the MT and CA at pH 7.4. The key experimental feature is that metalation of apo-MT with <4 mole equivalents Cd^2+^ at a pH < 7.4, results in predominant Cd_4_S_CYS-11_ clusters. The metalation rate of the clusters is shown in previous studies to be slower than the metalation of apo-MT to form beads [56].

### 4.7. Significance of MT–CA Interactions at Physiological pH and at Tumorigenic Extracellular Acidosis

As regulators of pH, CAs are hypothesized to play a vital role in the generation of the pH gradient associated with tumorigenesis and cancer progression. This gradient is characterized by mostly unchanged intracellular pH, and a sharp drop in extracellular pH to 6.5–7.1 [82]. This gradient is hypothesized to be achieved by the cooperation of intracellular CA-I and CA-II, and extracellular CA-IX and CA-XII. Extracellular CAs convert excess CO_2_ from highly metabolic cells such as cancer cells to HCO_3_^−^ and protons, allowing the HCO_3_^−^ to be pumped into the cell to maintain a stable intracellular pH while the remaining protons cause the shift to lower extracellular pH [21]. Alternatively, both intracellular and extracellular CAs may only play a role in buffering the pH changes produced by lactic acid from metabolic fermentation of cancer tissue, thus contributing to pH stabilization rather than direct acidosis [21].

The interaction between apo-MT and apo-CA at physiological pH must take place in order for CA to bind the essential Zn^2+^ needed for catalytic activity. At lower physiological pHs, this interaction is either very weak or does not occur, as the presence of apo-CA does not change the binding constants or metalation kinetics for apo-MT. Therefore, at a lower pH, such as the case in the extracellular matrix of cancerous tissue, MT may not be able to donate the catalytic Zn^2+^ to apo-CA as needed [84]. However, as stated above, apo-CA IX and XII are the active members of the CA family within tumors, and less so with CA II, but its activity can be directly affected by the cancer-induced change in the environment. If CA II is inactive partially because of the pH change and, therefore, lack of interaction with its metallochaperone, this would contribute to the abnormality of the cancerous tissue.

CA is negatively charged at physiological pH ~7, which can promote an attractive interaction between itself and the positively charged MT. However, at pH ~5, the CA is now also positively charged, which may repel the MT from CA, which would weaken the interaction between the two. For the protein–protein mechanism of Zn^2+^ metalation of CA, there must be an association between the proteins for proper Zn^2+^ transfer and subsequent Zn^2+^ metalation. Without effective metalation of CA by Zn^2+^, the enzymatic activity is stalled, and cellular homeostasis is disturbed. The acidic conditions of tumors can influence the protein–protein association and alters the metalation of CA, and thus the enzymatic activity of CA. This necessity for proper Zn^2+^ metalation of metalloproteins is noted in other cancer-related protein targets, namely matrix metalloproteases, histone deacetylases, zinc finger proteins, and tumor suppressor p53 [129], where the loss of zinc binding and the loss of effective MT docking contributes to carcinogenesis [130].

## 5. Conclusions

Bioinorganic chemistry is key to understanding the metallome. The metalation of metalloproteins is a key process that is still very poorly documented. In this review, we described a series of experiments concerning the metalation of carbonic anhydrase, using metallothionein as the donor. The data with Zn-MT and apo-CA clearly show that the individual binding constants of the MT and CA overlap only for the 6th and 7th Zn^2+^ bound. The change in the speciation for the first three zincs bound to MT suggested significant protein–protein interactions. Stopped-flow kinetics was used to provide further evidence of protein–protein interactions during metalation of apo-MT and apo-CA with Cd^2+^. The metalation properties of the apo-MT were significantly disturbed when the apo-MT metalated in the presence of apo-CA.

It has long been proposed that metallothionein acts as a metal donor for zinc enzymes. The results described in this review indicate that the metal donation pathway likely involves a protein–protein interaction, for which new methods are required to quantify. We look forward to advancements in mass spectrometry to provide such data.

## Abbreviations

ATP	Adenosine Triphophate
CA	Carbonic Anhydrase
CNS	Central Nervous System
ECM	Extracellular Matrix
EDTAH_4_	Ethylenediaminetetraacetic acid
ESI-MS	Electrospray Ionization Mass Spectrometry
IARC	International Agency for Research on Cancer
MRE	Metal Response Element
MT	Metallothionein
PG	Proteoglycan
